# Subject Index : Volume 10

**DOI:** 10.1111/jdi.13142

**Published:** 2019-11-03

1,25(OH)2D3

Vitamin D_3_ supplementation improves testicular function in diabetic rats through peroxisome proliferator‐activated receptor‐γ/transforming growth factor‐beta 1/nuclear factor‐kappa B, Liu 261–271

3‐carboxy‐4‐methyl‐5‐propyl‐2‐furanpropanoic acid

Serum 3‐carboxy‐4‐methyl‐5‐propyl‐2‐furanpropanoic acid is associated with lipid profiles and might protect against non‐alcoholic fatty liver disease in Chinese individuals, Dai 793–800

3T3‐L1 preadipocytes

*Kbtbd11* gene expression in adipose tissue increases in response to feeding and affects adipocyte differentiation, Watanabe 925–932

12(S)‐hydroxyeicosatetraenoic acid

12(S)‐hydroxyeicosatetraenoic acid impairs vascular endothelial permeability by altering adherens junction phosphorylation levels and affecting the binding and dissociation of its components in high glucose‐induced vascular injury, Wang 639–649

75‐g oral glucose tolerance test

Difference in the prevalence of gestational diabetes mellitus according to gestational age at 75‐g oral glucose tolerance test in Japan: The Japan Assessment of Gestational Diabetes Mellitus Screening trial, Iwama 1576–1585

abdominal ultrasound

Maturity‐onset diabetes of the young type 5 uncovered during pregnancy with a long‐term diagnosis of type 1 diabetes, Deng 1590–1592

access to care

Association between income levels and irregular physician visits after a health checkup, and its consequent effect on glycemic control among employees: A retrospective propensity score‐matched cohort study, Nishi 1372–1381

active glucagon‐like peptide‐1

Solid‐phase extraction treatment is required for measurement of active glucagon‐like peptide‐1 by enzyme‐linked immunosorbent assay kit affected by heterophilic antibodies, Hasegawa 302–308

acute inflammation

Drug fever and acute inflammation from hypercytokinemia triggered by dipeptidyl peptidase‐4 inhibitor vildagliptin, Anno 182–185

adenosine triphosphate‐sensitive potassium channel

Functional adenosine triphosphate‐sensitive potassium channel is required in high‐carbohydrate diet‐induced increase in β‐cell mass, Murase 238–250

adipocyte progenitors

M2‐like macrophages serve as a niche for adipocyte progenitors in adipose tissue, Nawaz 1394–1400

adipokine

Independent links between plasma xanthine oxidoreductase activity and levels of adipokines, Furuhashi 1059–1067

adipose tissue niche

M2‐like macrophages serve as a niche for adipocyte progenitors in adipose tissue, Nawaz 1394–1400

advanced glycation end‐products

Association of accumulated advanced glycation end‐products with a high prevalence of sarcopenia and dynapenia in patients with type 2 diabetes, Mori 1332–1340

adverse drug event

Safety and tolerability of empagliflozin in East Asian patients with type 2 diabetes: Pooled analysis of phase I–III clinical trials, Yabe 418–428

adverse event

Allergic contact dermatitis caused by isobornyl acrylate when using the FreeStyle^®^ Libre, Mine 1382–1384

affinity

Discrepancy of glutamic acid decarboxylase 65 autoantibody results between RSR radioimmunoassay and enzyme‐linked immunosorbent assay in patients with type 1 diabetes is related to autoantibody affinity, Kawasaki 990–996

alanine aminotransferase

Association of gamma‐glutamyl transferase and alanine aminotransferase with type 2 diabetes mellitus incidence in middle‐aged Japanese men: 12‐year follow up, Kaneko 837–845

albuminuria

Secular changes in clinical manifestations of kidney disease among Japanese adults with type 2 diabetes from 1996 to 2014, Kume 1032–1040

alendronate

Alendronate improves fasting plasma glucose and insulin sensitivity, and decreases insulin resistance in prediabetic osteopenic postmenopausal women: A randomized triple‐blind clinical trial, Karimi Fard 731–737

amino acids

Plasma tyrosine and its interaction with low high‐density lipoprotein cholesterol and the risk of type 2 diabetes mellitus in Chinese, Li 491–498

antidiabetic agents

Effects of liraglutide, metformin and gliclazide on body composition in patients with both type 2 diabetes and non‐alcoholic fatty liver disease: A randomized trial, Feng 399–407

anti‐glutamic acid decarboxylase antibody

Actual condition survey regarding mismatch of measurements between radioimmunoassay and enzyme‐linked immunosorbent assay tests for anti‐glutamic acid decarboxylase antibody in real‐world clinical practice, Oikawa 685–689
Discrepancy of glutamic acid decarboxylase 65 autoantibody results between RSR radioimmunoassay and enzyme‐linked immunosorbent assay in patients with type 1 diabetes is related to autoantibody affinity, Kawasaki 990–996

anti‐programmed death‐ligand 1 antibody

Case of fulminant type 1 diabetes induced by the anti‐programmed death‐ligand 1 antibody, avelumab, Shibayama 1385–1387

aortic root dilatation

Diabetes attenuated age‐related aortic root dilatation in end‐stage renal disease patients receiving peritoneal dialysis, Ye 1550–1557

apoptosis

*Polygonatum sibiricum *polysaccharide potentially attenuates diabetic retinal injury in a diabetic rat model, Wang 915–924

appetite

Effects of ipragliflozin on glycemic control, appetite and its related hormones: A prospective, multicenter, open‐label study (SOAR‐KOBE Study), Miura 1254–1261

Asians

Basal insulin therapy: Unmet medical needs in Asia and the new insulin glargine in diabetes treatment, Tien 560–570

autophagy

p62‐mediated autophagy affects nutrition‐dependent insulin receptor substrate 1 dynamics in 3T3‐L1 preadipocytes, Igawa 32–42
Fat‐specific protein 27α inhibits autophagy‐dependent lipid droplet breakdown in white adipocytes, Nakajima 1419–1429

β‐cell

Leucine–glycine and carnosine dipeptides prevent diabetes induced by multiple low‐doses of streptozotocin in an experimental model of adult mice, Vahdatpour 1177–1188

β‐cell mass

Functional adenosine triphosphate‐sensitive potassium channel is required in high‐carbohydrate diet‐induced increase in β‐cell mass, Murase 238–250

*BACH2*

Variants in the *BACH2* and *CLEC16A* gene might be associated with susceptibility to insulin‐triggered type 1 diabetes, Onuma 1447–1453

basal–bolus therapy

Comparison of the efficacy and safety of insulin degludec/aspart (twice‐daily injections), insulin glargine 300 U/mL, and insulin glulisine (basal–bolus therapy), Kawaguchi 1527–1536

bioinformatics analysis

Identification of key genes and pathways in diabetic nephropathy by bioinformatics analysis, Geng 972–984

birthweight

Being underweight in adolescence is independently associated with adult‐onset diabetes among women: The Japan Nurses’ Health Study, Katanoda 827–836
Weight control before and during pregnancy for patients with gestational diabetes mellitus, Yasuda 1075–1082

blood glucose

Understanding of antidiabetic medication is associated with blood glucose in patients with type 2 diabetes: At baseline date of the KAMOGAWA‐DM cohort study, Sakai 458–465

body composition

Effects of liraglutide, metformin and gliclazide on body composition in patients with both type 2 diabetes and non‐alcoholic fatty liver disease: A randomized trial, Feng 399–407

body fat percentage

Importance of physical evaluation using skeletal muscle mass index and body fat percentage to prevent sarcopenia in elderly Japanese diabetes patients, Fukuoka 322–330

body mass index

Combined aerobic and resistance training, and incidence of diabetes: A retrospective cohort study in Japanese older women, Sawada 997–1003
Impact of body mass index on the efficacy and safety of ipragliflozin in Japanese patients with type 2 diabetes mellitus: A subgroup analysis of 3‐month interim results from the Specified Drug Use Results Survey of Ipragliflozin Treatment in Type 2 Diabetic Patients: Long‐term Use study, Tobe 1262–1271
Lower body mass index is not of more benefit for diabetic complications, Zhang 1307–1317

bodyweight

Ipragliflozin, a sodium–glucose cotransporter 2 inhibitor, reduces bodyweight and fat mass, but not muscle mass, in Japanese type 2 diabetes patients treated with insulin: A randomized clinical trial, Inoue 1012–1021

bodyweight changes

Being underweight in adolescence is independently associated with adult‐onset diabetes among women: The Japan Nurses’ Health Study, Katanoda 827–836

bolus doses

Assessment of optimal insulin administration timing for standard carbohydrates‐rich meals using continuous glucose monitoring in children with type 1 diabetes: A cross‐over randomized study, Tucholski 1237–1245

brown rice

Brown rice‐specific γ‐oryzanol as a promising prophylactic avenue to protect against diabetes mellitus and obesity in humans, Masuzaki 18–25

bullous pemphigoid

Dipeptidyl peptidase‐4 inhibitors‐associated bullous pemphigoid: A retrospective study of 168 pemphigoid and 9,304 diabetes mellitus patients, Kawaguchi 392–398

calcium sensor

Protein kinase C iota facilitates insulin‐induced glucose transport by phosphorylation of soluble nSF attachment protein receptor regulator (SNARE) double C2 domain protein b, Nomiyama 591–601

carbohydrate

Functional adenosine triphosphate‐sensitive potassium channel is required in high‐carbohydrate diet‐induced increase in β‐cell mass, Murase 238–250

cardiometabolic risk factor

Cardiometabolic risk factors in Thai individuals with prediabetes treated in a high‐risk, prevention clinic: Unexpected relationship between high‐density lipoprotein cholesterol and glycemia in men, Srivanichakorn 771–779

cardiorenal syndrome

Canagliflozin, a sodium–glucose cotransporter 2 inhibitor, normalizes renal susceptibility to type 1 cardiorenal syndrome through reduction of renal oxidative stress in diabetic rats, Kimura 933–946

cardiovascular risk

Metabolic syndrome increases cardiovascular risk in a population with prediabetes: A prospective study in a cohort of Chinese adults, Chen 673–679

cardiovascular risk factors

Effects of sodium–glucose cotransporter 2 inhibitors in addition to insulin therapy on cardiovascular risk factors in type 2 diabetes patients: A meta‐analysis of randomized controlled trials, Wu 446–457

carotid ultrasound

Clinical utility of carotid ultrasonography: Application for the management of patients with diabetes, Katakami 883–898

case–control study

Exploration of predictive metabolic factors for gestational diabetes mellitus in Japanese women using metabolomic analysis, Sakurai 513–520

catastrophic health expenditure

Catastrophic health expenditure among type 2 diabetes mellitus patients: A province‐wide study in Shandong, China, Jing 283–289

CD4^+^ T lymphocytes

Elevated histone H3 acetylation is associated with genes involved in T lymphocyte activation and glutamate decarboxylase antibody production in patients with type 1 diabetes, Wang 51–61

China

Prevalence of gestational diabetes mellitus in mainland China: A systematic review and meta‐analysis, Gao 154–162
Direct medical costs for patients with type 2 diabetes in 16 tertiary hospitals in urban China: A multicenter prospective cohort study, Li 539–551

chronic complications

Genetic variants in *DNMT1 *and the risk of cardiac autonomic neuropathy in women with type 1 diabetes, Santos‐Bezerra 985–989

chronic kidney disease

Self‐reported snoring is associated with chronic kidney disease independent of metabolic syndrome in middle‐aged and elderly Chinese, Song 124–130

cigarette smoking

Review of the role of cigarette smoking in diabetic foot, Xia 202–215

*CLEC16A*

Variants in the *BACH2* and *CLEC16A* gene might be associated with susceptibility to insulin‐triggered type 1 diabetes, Onuma 1447–1453

clinical characteristics

Painful and non‐painful diabetic polyneuropathy: Clinical characteristics and diagnostic issues, Gylfadottir 1148–1157

clinical practice pattern

Clinical preferences and trends of anti‐vascular endothelial growth factor treatments for diabetic macular edema in Japan, Sugimoto 475–483

co‐expression network

Key genes and co‐expression network analysis in the livers of type 2 diabetes patients, Li 951–962

cognitive function

Serum high‐density lipoprotein cholesterol is a protective predictor of executive function in older patients with diabetes mellitus, Sun 139–146

Commentary

Alpha the versatile: Guardians of the islets, Kawamori 26–28
Obesity‐induced insulin resistance and macrophage infiltration of the adipose tissue: A vicious cycle, Lee 29–31
Unappreciated role of low‐density lipoprotein receptor‐related protein 1 in pancreatic β cells: Multiple roles of low‐density lipoprotein receptor‐related protein 1 in glucose and lipid metabolism, Watada 216–218
New risk factors of severe hypoglycemia, Matsuhisa 219–220
Fat cell lipolysis and future weight gain, Nishizawa 221–223
Human glomerular transcriptome of diabetic kidneys: Can the podocyte cytoskeleton be a therapeutic target?, Maezawa 224–226
Different trends in causes of death in patients with diabetes between Japan and the USA, Tsunekawa 571–573
Neuronatin and glucose‐induced stress in pancreatic β‐cells, Asahara 574–576
Aspirin for primary prevention of cardiovascular disease in diabetes, Xing 899–901
Twincretin as a potential therapeutic for the management of type 2 diabetes with obesity, Usui 902–905
Rho‐associated, coiled‐coil‐containing protein kinase 1 as a new player in the regulation of hepatic lipogenesis, Miyamoto 1165–1167
Bullous pemphigoid with dipeptidyl peptidase‐4 inhibitors: Clinical features and pathophysiology, Murakami 1168–1170
Novel mechanism of impaired metabolism‐secretion coupling in β‐cells: Loss of cytosolic adenosine triphosphate by leakage, Fujimoto 1401–1404

complications

Direct medical cost of diabetes in rural China using electronic insurance claims data and diabetes management data, Wu 531–538

continuous glucose monitoring

Which is better, high‐dose metformin monotherapy or low‐dose metformin/linagliptin combination therapy, in improving glycemic variability in type 2 diabetes patients with insufficient glycemic control despite low‐dose metformin monotherapy? A randomized, cross‐over, continuous glucose monitoring‐based pilot study, Takahashi 714–722
Accuracy of flash glucose monitoring in insulin‐treated patients with type 2 diabetes, Sato 846–850

core item sets

Recommended configuration for personal health records by standardized data item sets for diabetes mellitus and associated chronic diseases: A report from Collaborative Initiative by six Japanese Associations, Nakashima 868–875

cultural context

Novel psychosocial factor involved in diabetes self‐care in the Japanese cultural context, Mano 1102–1107

C‐X‐C motif chemokine 10

Circulating anti‐glutamic acid decarboxylase‐65 antibody titers are positively associated with the capacity of insulin secretion in acute‐onset type 1 diabetes with short duration in a Japanese population, Yamamura 1480–1489

decision support system

Feasibility and safety of using an automated decision support system for insulin therapy in the treatment of steroid‐induced hyperglycemia in patients with acute graft‐versus‐host disease: A randomized trial, Aberer 339–342

dental pulp stem cell

Conditioned media from dental pulp stem cells improved diabetic polyneuropathy through anti‐inflammatory, neuroprotective and angiogenic actions: Cell‐free regenerative medicine for diabetic polyneuropathy, Makino 1199–1208

depression

Longitudinal investigation of the reciprocal relationship between depressive symptoms and glycemic control: The moderation effects of sex and perceived support, Chiu 801–808

deprivation

Type 2 diabetes mellitus and neighborhood deprivation index: A spatial analysis in Zhejiang, China, Zhang 272–282

determinants

Catastrophic health expenditure among type 2 diabetes mellitus patients: A province‐wide study in Shandong, China, Jing 283–289

diabetes

Contribution and interaction of the low‐density lipoprotein cholesterol to high‐density lipoprotein cholesterol ratio and triglyceride to diabetes in hypertensive patients: A cross‐sectional study, Hong 131–138
Vitamin D_3_ supplementation improves testicular function in diabetic rats through peroxisome proliferator‐activated receptor‐γ/transforming growth factor‐beta 1/nuclear factor‐kappa B, Liu 261–271
Type 2 diabetes mellitus and neighborhood deprivation index: A spatial analysis in Zhejiang, China, Zhang 272–282
Impacts of the 2016 Kumamoto Earthquake on glycemic control in patients with diabetes, Kondo 521–530
Direct medical cost of diabetes in rural China using electronic insurance claims data and diabetes management data, Wu 531–538
Basal insulin therapy: Unmet medical needs in Asia and the new insulin glargine in diabetes treatment, Tien 560–570
Effect of oral magnesium sulfate administration on lectin‐like oxidized low‐density lipoprotein receptor‐1 gene expression to prevent atherosclerosis in diabetic rat vessels, Fazlali 650–658
Prevalence of diabetes and its effects on stroke outcomes: A meta‐analysis and literature review, Lau 780–792
*Helicobacter pylori *eradication improves glycemic control in type 2 diabetes patients with asymptomatic active *Helicobacter pylori *infection, Cheng 1092–1101
Diabetes Care Providers’ Manual for Disaster Diabetes Care, Satoh 1118–1142
Low serum creatinine and risk of diabetes: The Japan Epidemiology Collaboration on Occupational Health Study, Hu 1209–1214
Associations between stressful life events and diabetes: Findings from the China Kadoorie Biobank study of 500,000 adults, Wang 1215–1222
Changes in soluble tumor necrosis factor receptor type 1 levels and early renal function decline in patients with diabetes, MacIsaac 1537–1542
Diabetes attenuated age‐related aortic root dilatation in end‐stage renal disease patients receiving peritoneal dialysis, Ye 1550–1557

diabetes mellitus

G protein‐coupled receptor 119 agonist DS‐8500a effects on pancreatic β‐cells in Japanese type 2 diabetes mellitus patients, Watada 84–93
Serum high‐density lipoprotein cholesterol is a protective predictor of executive function in older patients with diabetes mellitus, Sun 139–146
Prevalence of diabetes among homeless men in Nagoya, Japan: A survey study, Yamamoto 667–672
Accuracy of flash glucose monitoring in insulin‐treated patients with type 2 diabetes, Sato 846–850
Clinical utility of carotid ultrasonography: Application for the management of patients with diabetes, Katakami 883–898
Associations of nutrient intakes with obesity and diabetes mellitus in the longitudinal medical surveys of Japanese Americans, Sugihiro 1229–1236

diabetic cardiomyopathy

Restoration of myocardial glucose uptake with facilitated myocardial glucose transporter 4 translocation contributes to alleviation of diabetic cardiomyopathy in rats after duodenal‐jejunal bypass, Huang 626–638

diabetic complication

Discordance in risk factors for the progression of diabetic retinopathy and diabetic nephropathy in patients with type 2 diabetes mellitus, Song 745–752

diabetic foot ulcers

Review of the role of cigarette smoking in diabetic foot, Xia 202–215

diabetic kidney disease

Effect of switching to teneligliptin from other dipeptidyl peptidase‐4 inhibitors on glucose control and renoprotection in type 2 diabetes patients with diabetic kidney disease, Kitada 706–713
Empagliflozin and kidney outcomes in Asian patients with type 2 diabetes and established cardiovascular disease: Results from the EMPA‐REG OUTCOME^®^ trial, Kadowaki 760–770
Secular changes in clinical manifestations of kidney disease among Japanese adults with type 2 diabetes from 1996 to 2014, Kume 1032–1040

diabetic macular edema

Clinical preferences and trends of anti‐vascular endothelial growth factor treatments for diabetic macular edema in Japan, Sugimoto 475–483

diabetic nephropathy

Protein kinase C and protein kinase A are involved in the protection of recombinant human glucagon‐like peptide‐1 on glomeruli and tubules in diabetic rats, Yin 613–625
Pathological significance of urinary complement activation in diabetic nephropathy: A full view from the development of the disease, Zheng 738–744
Discordance in risk factors for the progression of diabetic retinopathy and diabetic nephropathy in patients with type 2 diabetes mellitus, Song 745–752
Identification of key genes and pathways in diabetic nephropathy by bioinformatics analysis, Geng 972–984
Secular changes in clinical manifestations of kidney disease among Japanese adults with type 2 diabetes from 1996 to 2014, Kume 1032–1040
Association of renal arteriosclerosis and hypertension with renal and cardiovascular outcomes in Japanese type 2 diabetes patients with diabetic nephropathy, Shimizu 1041–1049
Maximum morning home systolic blood pressure is an indicator of the development of diabetic nephropathy: The KAMOGAWA‐HBP study, Okamura 1543–1549

diabetic neuropathy

Omega‐3 polyunsaturated fatty acids exert anti‐oxidant effects through the nuclear factor (erythroid‐derived 2)‐related factor 2 pathway in immortalized mouse Schwann cells, Tatsumi 602–612
Painful and non‐painful diabetic polyneuropathy: Clinical characteristics and diagnostic issues, Gylfadottir 1148–1157

Conditioned media from dental pulp stem cells improved diabetic polyneuropathy through anti‐inflammatory, neuroprotective and angiogenic actions: Cell‐free regenerative medicine for diabetic polyneuropathy, Makino 1199–1208

diabetic peripheral neuropathic pain

Mirogabalin for the treatment of diabetic peripheral neuropathic pain: A randomized, double‐blind, placebo‐controlled phase III study in Asian patients, Baba 1299–1306

diabetic polyneuropathy

Aldose reductase inhibitor ranirestat significantly improves nerve conduction velocity in diabetic polyneuropathy: A randomized double‐blind placebo‐controlled study in Japan, Sekiguchi 466–474
Validity and reliability of a point‐of‐care nerve conduction device in diabetes patients, Shibata 1291–1298

diabetic retinopathy

(Pro)renin receptor: Involvement in diabetic retinopathy and development of molecular targeted therapy, Kanda 6–17
Discordance in risk factors for the progression of diabetic retinopathy and diabetic nephropathy in patients with type 2 diabetes mellitus, Song 745–752
Glycemic variability assessed by continuous glucose monitoring and the risk of diabetic retinopathy in latent autoimmune diabetes of the adult and type 2 diabetes, Lu 753–759
*Polygonatum sibiricum *polysaccharide potentially attenuates diabetic retinal injury in a diabetic rat model, Wang 915–924

diagnosis

Painful and non‐painful diabetic polyneuropathy: Clinical characteristics and diagnostic issues, Gylfadottir 1148–1157

dietary carbohydrate

Dietary survey in Japanese patients with type 2 diabetes and the influence of dietary carbohydrate on glycated hemoglobin: The Sleep and Food Registry in Kanagawa study, Yamakawa 309–317

differentially expressed genes

Identification of key genes and pathways in diabetic nephropathy by bioinformatics analysis, Geng 972–984

differentiation

*Kbtbd11* gene expression in adipose tissue increases in response to feeding and affects adipocyte differentiation, Watanabe 925–932

dipeptidyl peptidase‐4 inhibitor

Altered T‐cell subsets and transcription factors in latent autoimmune diabetes in adults taking sitagliptin, a dipeptidyl peptidase‐4 inhibitor: A 1‐year open‐label randomized controlled trial, Wang 375–382
Which is better, high‐dose metformin monotherapy or low‐dose metformin/linagliptin combination therapy, in improving glycemic variability in type 2 diabetes patients with insufficient glycemic control despite low‐dose metformin monotherapy? A randomized, cross‐over, continuous glucose monitoring‐based pilot study, Takahashi 714–722
Effects of mobile phone application combined with or without self‐monitoring of blood glucose on glycemic control in patients with diabetes: A randomized controlled trial, Yu 1365–1371

dipeptidyl peptidase‐4 inhibitors

Dipeptidyl peptidase‐4 inhibitors‐associated bullous pemphigoid: A retrospective study of 168 pemphigoid and 9,304 diabetes mellitus patients, Kawaguchi 392–398
Effects of metformin and alogliptin on body composition in people with type 2 diabetes, Takeshita 723–730

direct medical cost

Direct medical cost of diabetes in rural China using electronic insurance claims data and diabetes management data, Wu 531–538

direct medical costs

Direct medical costs for patients with type 2 diabetes in 16 tertiary hospitals in urban China: A multicenter prospective cohort study, Li 539–551

disaster

Diabetes Care Providers’ Manual for Disaster Diabetes Care, Satoh 1118–1142

drug fever

Drug fever and acute inflammation from hypercytokinemia triggered by dipeptidyl peptidase‐4 inhibitor vildagliptin, Anno 182–185

drug side‐effect

Dipeptidyl peptidase‐4 inhibitors‐associated bullous pemphigoid: A retrospective study of 168 pemphigoid and 9,304 diabetes mellitus patients, Kawaguchi 392–398

dual‐energy X‐ray absorption

Sodium–glucose cotransporter 2 inhibitor‐induced changes in body composition and simultaneous changes in metabolic profile: 52‐week prospective LIGHT (Luseogliflozin: the Components of Weight Loss in Japanese Patients with Type 2 Diabetes Mellitus) Study, Sasaki 108–117

dulaglutide

Improvement in treatment satisfaction after switching from liraglutide to dulaglutide in patients with type 2 diabetes: A randomized controlled trial, Takase 699–705

duodenal‐jejunal bypass

Restoration of myocardial glucose uptake with facilitated myocardial glucose transporter 4 translocation contributes to alleviation of diabetic cardiomyopathy in rats after duodenal‐jejunal bypass, Huang 626–638

dynapenia

Association of accumulated advanced glycation end‐products with a high prevalence of sarcopenia and dynapenia in patients with type 2 diabetes, Mori 1332–1340

early‐onset diabetes

Identification of a variant associated with early‐onset diabetes in the intron of the insulin gene with exome sequencing, Matsuno 947–950

earthquake

Impacts of the 2016 Kumamoto Earthquake on glycemic control in patients with diabetes, Kondo 521–530

Editorial

Gastric inhibitory polypeptide/glucose‐dependent insulinotropic polypeptide signaling in adipose tissue, Yamane 3–5
Sarcopenia, frailty circle and treatment with sodium–glucose cotransporter 2 inhibitors, Sasaki 193–195
Cardiorenal protective effect of sodium–glucose cotransporter 2 inhibitors and mitochondrial function, Lee 557–559
Sodium–glucose cotransporter 2 inhibitor‐associated diabetic ketoacidosis in patients with type 1 diabetes: Metabolic imbalance as an underlying mechanism, Ogawa 879–882
A new door opens, but it is essential to accumulate further clinical evidence to control heart failure in diabetes with preserved ejection fraction, Kashiwagi 1145–1147
Glutaminostatin: Another facet of glucagon as a regulator of plasma amino acid concentrations, Hayashi 1391–1393

elderly

Reduction in glucose fluctuations in elderly patients with type 2 diabetes using repaglinide: A randomized controlled trial of repaglinide vs sulfonylurea, Omori 367–374

elderly diabetes

Importance of physical evaluation using skeletal muscle mass index and body fat percentage to prevent sarcopenia in elderly Japanese diabetes patients, Fukuoka 322–330

empagliflozin

Empagliflozin and kidney outcomes in Asian patients with type 2 diabetes and established cardiovascular disease: Results from the EMPA‐REG OUTCOME^®^ trial, Kadowaki 760–770

endothelial permeability

12(S)‐hydroxyeicosatetraenoic acid impairs vascular endothelial permeability by altering adherens junction phosphorylation levels and affecting the binding and dissociation of its components in high glucose‐induced vascular injury, Wang 639–649

end‐stage renal disease

Diabetes attenuated age‐related aortic root dilatation in end‐stage renal disease patients receiving peritoneal dialysis, Ye 1550–1557

enzyme kinetics

Identification and functional analysis of *GCK *gene mutations in 12 Chinese families with hyperglycemia, Wang 963–971
Clinical and enzymatic phenotypes in congenital hyperinsulinemic hypoglycemia due to glucokinase‐activating mutations: A report of two cases and a brief overview of the literature, Ping 1454–1462

enzyme‐linked immunosorbent assay

Actual condition survey regarding mismatch of measurements between radioimmunoassay and enzyme‐linked immunosorbent assay tests for anti‐glutamic acid decarboxylase antibody in real‐world clinical practice, Oikawa 685–689
Discrepancy of glutamic acid decarboxylase 65 autoantibody results between RSR radioimmunoassay and enzyme‐linked immunosorbent assay in patients with type 1 diabetes is related to autoantibody affinity, Kawasaki 990–996

epidemiology

Objectively measured sedentary time and diabetes mellitus in a general Japanese population: The Hisayama Study, Honda 809–816
Combined aerobic and resistance training, and incidence of diabetes: A retrospective cohort study in Japanese older women, Sawada 997–1003

epigenetics

Genetic variants in *DNMT1 *and the risk of cardiac autonomic neuropathy in women with type 1 diabetes, Santos‐Bezerra 985–989

eradication

Association between *Helicobacter pylori *infection, eradication and diabetes mellitus, Kato 1341–1346

estimated glomerular filtration rate

Protective effect of sodium–glucose cotransporter 2 inhibitors in patients with rapid renal function decline, stage G3 or G4 chronic kidney disease and type 2 diabetes, Miyoshi 1510–1517

exercise

Combined aerobic and resistance training, and incidence of diabetes: A retrospective cohort study in Japanese older women, Sawada 997–1003

family history of diabetes

High, but stable, trend in the prevalence of gestational diabetes mellitus: A population‐based study in Xiamen, China, Yan 1358–1364

family support

Longitudinal investigation of the reciprocal relationship between depressive symptoms and glycemic control: The moderation effects of sex and perceived support, Chiu 801–808

fast muscle fibers

Effects of low‐intensity resistance training on muscular function and glycemic control in older adults with type 2 diabetes, Takenami 331–338

fat‐specific protein 27

Fat‐specific protein 27α inhibits autophagy‐dependent lipid droplet breakdown in white adipocytes, Nakajima 1419–1429

feasibility trial

Feasibility and safety of using an automated decision support system for insulin therapy in the treatment of steroid‐induced hyperglycemia in patients with acute graft‐versus‐host disease: A randomized trial, Aberer 339–342

fibroblast growth factor 19

Decreased placental and muscular expression of the fibroblast growth factor 19 in gestational diabetes mellitus, Wang 171–181

fibrosis

Nicotinic alpha‐7 acetylcholine receptor deficiency exacerbates hepatic inflammation and fibrosis in a mouse model of non‐alcoholic steatohepatitis, Kimura 659–666

flash glucose monitoring

Comparison of insulin glargine 300 U/mL and insulin degludec using flash glucose monitoring: A randomized cross‐over study, Yamabe 352–357
Accuracy of flash glucose monitoring in insulin‐treated patients with type 2 diabetes, Sato 846–850

free fatty acid

Interactive association of lipopolysaccharide and free fatty acid with the prevalence of type 2 diabetes: A community‐based cross‐sectional study, Huang 1438–1446

FreeStyle^®^ Libre

Allergic contact dermatitis caused by isobornyl acrylate when using the FreeStyle^®^ Libre, Mine 1382–1384

fulminant type 1 diabetes

Pathogenesis of fulminant type 1 diabetes: Genes, viruses and the immune mechanism, and usefulness of patient‐derived induced pluripotent stem cells for future research, Hosokawa 1158–1164
Case of fulminant type 1 diabetes induced by the anti‐programmed death‐ligand 1 antibody, avelumab, Shibayama 1385–1387

G0/G1 switch 2 gene

Soluble CD163 correlates with lipid metabolic adaptations in type 1 diabetes patients during ketoacidosis, Svart 67–72

γ‐oryzanol

Brown rice‐specific γ‐oryzanol as a promising prophylactic avenue to protect against diabetes mellitus and obesity in humans, Masuzaki 18–25

gamma‐glutamyl transferase

Association of gamma‐glutamyl transferase and alanine aminotransferase with type 2 diabetes mellitus incidence in middle‐aged Japanese men: 12‐year follow up, Kaneko 837–845

gastric emptying

Efficacy of metformin on postprandial plasma triglyceride concentration by administration timing in patients with type 2 diabetes mellitus: A randomized cross‐over pilot study, Sato 1284–1290

gastric inhibitory polypeptide

Free fatty acid receptors, G protein‐coupled receptor 120 and G protein‐coupled receptor 40, are essential for oil‐induced gastric inhibitory polypeptide secretion, Sankoda 1430–1437

genital infection

Safety and tolerability of empagliflozin in East Asian patients with type 2 diabetes: Pooled analysis of phase I–III clinical trials, Yabe 418–428

gestational age

Difference in the prevalence of gestational diabetes mellitus according to gestational age at 75‐g oral glucose tolerance test in Japan: The Japan Assessment of Gestational Diabetes Mellitus Screening trial, Iwama 1576–1585

gestational diabetes

Clinical and genetic characteristics of abnormal glucose tolerance in Japanese women in the first year after gestational diabetes mellitus, Kasuga 817–826

gestational diabetes mellitus

Prevalence of gestational diabetes mellitus in mainland China: A systematic review and meta‐analysis, Gao 154–162
Effects of probiotic supplements on insulin resistance in gestational diabetes mellitus: A double‐blind randomized controlled trial, Kijmanawat 163–170
Decreased placental and muscular expression of the fibroblast growth factor 19 in gestational diabetes mellitus, Wang 171–181
Downregulation of peroxisome proliferator‐activated receptor gamma in the placenta correlates to hyperglycemia in offspring at young adulthood after exposure to gestational diabetes mellitus, Zhao 499–512
Exploration of predictive metabolic factors for gestational diabetes mellitus in Japanese women using metabolomic analysis, Sakurai 513–520
Weight control before and during pregnancy for patients with gestational diabetes mellitus, Yasuda 1075–1082
Influence of different diagnostic criteria on gestational diabetes mellitus incidence and medical expenditures in China, He 1347–1357
High, but stable, trend in the prevalence of gestational diabetes mellitus: A population‐based study in Xiamen, China, Yan 1358–1364
Difference in the prevalence of gestational diabetes mellitus according to gestational age at 75‐g oral glucose tolerance test in Japan: The Japan Assessment of Gestational Diabetes Mellitus Screening trial, Iwama 1576–1585

glargine 300

Efficacy and safety of insulin glargine 300 U/mL vs insulin degludec in patients with type 2 diabetes: A randomized, open‐label, cross‐over study using continuous glucose monitoring profiles, Kawaguchi 343–351

glucagon

Dysregulated plasma glucagon levels in Japanese young adult type 1 diabetes patients, Kawamori 62–66

glucagon‐like peptide‐1

Glucagon‐like peptide‐1 receptor agonists for type 2 diabetes: A rational drug development, Ahrén 196–201

glucagon‐like peptide‐1 receptor

Long‐term safety and efficacy of the sodium–glucose cotransporter 2 inhibitor, tofogliflozin, added on glucagon‐like peptide‐1 receptor agonist in Japanese patients with type 2 diabetes mellitus: A 52‐week open‐label, multicenter, post‐marketing clinical study, Terauchi 1518–1526

glucagon‐like peptide‐1 receptor agonists

Clinical factors associated with the occurrence of nausea and vomiting in type 2 diabetes patients treated with glucagon‐like peptide‐1 receptor agonists, Shiomi 408–417

glucokinase

Identification and functional analysis of *GCK *gene mutations in 12 Chinese families with hyperglycemia, Wang 963–971
Clinical and enzymatic phenotypes in congenital hyperinsulinemic hypoglycemia due to glucokinase‐activating mutations: A report of two cases and a brief overview of the literature, Ping 1454–1462
Pregnancy outcome of Japanese patients with glucokinase–maturity‐onset diabetes of the young, Hosokawa 1586–1589

glucose

Leucine–glycine and carnosine dipeptides prevent diabetes induced by multiple low‐doses of streptozotocin in an experimental model of adult mice, Vahdatpour 1177–1188

glucose‐dependent insulinotropic polypeptide

Glucose‐dependent insulinotropic polypeptide deficiency reduced fat accumulation and insulin resistance, but deteriorated bone loss in ovariectomized mice, Shimazu‐Kuwahara 909–914

glucose tolerance test

Clinical and genetic characteristics of abnormal glucose tolerance in Japanese women in the first year after gestational diabetes mellitus, Kasuga 817–826

glucose transporter 4

Protein kinase C iota facilitates insulin‐induced glucose transport by phosphorylation of soluble nSF attachment protein receptor regulator (SNARE) double C2 domain protein b, Nomiyama 591–601

glucose uptake

Restoration of myocardial glucose uptake with facilitated myocardial glucose transporter 4 translocation contributes to alleviation of diabetic cardiomyopathy in rats after duodenal‐jejunal bypass, Huang 626–638

glucose variability

Reduction in glucose fluctuations in elderly patients with type 2 diabetes using repaglinide: A randomized controlled trial of repaglinide vs sulfonylurea, Omori 367–374

glutamic acid decarboxylase antibody

Maturity‐onset diabetes of the young type 5 uncovered during pregnancy with a long‐term diagnosis of type 1 diabetes, Deng 1590–1592

glutamic decarboxylase‐65

Circulating anti‐glutamic acid decarboxylase‐65 antibody titers are positively associated with the capacity of insulin secretion in acute‐onset type 1 diabetes with short duration in a Japanese population, Yamamura 1480–1489

glycated hemoglobin

Dietary survey in Japanese patients with type 2 diabetes and the influence of dietary carbohydrate on glycated hemoglobin: The Sleep and Food Registry in Kanagawa study, Yamakawa 309–317
Effects of low‐intensity resistance training on muscular function and glycemic control in older adults with type 2 diabetes, Takenami 331–338
Should sulfonylurea be discontinued or maintained at the lowest dose when starting ipragliflozin? A multicenter observational study in Japanese patients with type 2 diabetes, Takahashi 429–438

glycemia

Cardiometabolic risk factors in Thai individuals with prediabetes treated in a high‐risk, prevention clinic: Unexpected relationship between high‐density lipoprotein cholesterol and glycemia in men, Srivanichakorn 771–779

glycemic control

Impacts of the 2016 Kumamoto Earthquake on glycemic control in patients with diabetes, Kondo 521–530
Longitudinal investigation of the reciprocal relationship between depressive symptoms and glycemic control: The moderation effects of sex and perceived support, Chiu 801–808
*Helicobacter pylori *eradication improves glycemic control in type 2 diabetes patients with asymptomatic active *Helicobacter pylori *infection, Cheng 1092–1101
Association between income levels and irregular physician visits after a health checkup, and its consequent effect on glycemic control among employees: A retrospective propensity score‐matched cohort study, Nishi 1372–1381

glycemic variability

Glycemic variability assessed by continuous glucose monitoring and the risk of diabetic retinopathy in latent autoimmune diabetes of the adult and type 2 diabetes, Lu 753–759

glycine

Glycine increases glyoxalase‐1 function by promoting nuclear factor erythroid 2‐related factor 2 translocation into the nucleus of kidney cells of streptozotocin‐induced diabetic rats, Wang 1189–1198

glycosylated hemoglobin

Long working hours and skipping breakfast concomitant with late evening meals are associated with suboptimal glycemic control among young male Japanese patients with type 2 diabetes, Azami 73–83

glyoxalase‐1

Glycine increases glyoxalase‐1 function by promoting nuclear factor erythroid 2‐related factor 2 translocation into the nucleus of kidney cells of streptozotocin‐induced diabetic rats, Wang 1189–1198

G protein‐coupled receptor 40

Free fatty acid receptors, G protein‐coupled receptor 120 and G protein‐coupled receptor 40, are essential for oil‐induced gastric inhibitory polypeptide secretion, Sankoda 1430–1437

G protein‐coupled receptor 120

Free fatty acid receptors, G protein‐coupled receptor 120 and G protein‐coupled receptor 40, are essential for oil‐induced gastric inhibitory polypeptide secretion, Sankoda 1430–1437

gut microbiota

Alteration of gut microbiota by a Westernized lifestyle and its correlation with insulin resistance in non‐diabetic Japanese men, Yamashita 1463–1470

heart rate

Independent and interactive associations of heart rate and body mass index or blood pressure with type 2 diabetes mellitus incidence: A prospective cohort study, Xu 1068–1074

*Helicobacter* eradication

*Helicobacter pylori *eradication improves glycemic control in type 2 diabetes patients with asymptomatic active *Helicobacter pylori *infection, Cheng 1092–1101

*Helicobacter pylori*

Association between *Helicobacter pylori *infection, eradication and diabetes mellitus, Kato 1341–1346

hepatic fat fraction

Effects of canagliflozin on body composition and hepatic fat content in type 2 diabetes patients with non‐alcoholic fatty liver disease, Inoue 1004–1011

hepatocyte growth factor

Hepatocyte growth factor alleviates hepatic insulin resistance and lipid accumulation in high‐fat diet‐fed mice, Jing 251–260

hepatocyte nuclear factor 1β gene

Phenotypic differences and similarities of monozygotic twins with maturity‐onset diabetes of the young type 5, Ohara 1112–1115

hepatocyte nuclear factor 4α

*In silico* and *in vitro* analyses of the pathological relevance of the R258H mutation of hepatocyte nuclear factor 4α identified in maturity‐onset diabetes of the young type 1, Sugawara 680–684

high‐density lipoprotein cholesterol

Serum high‐density lipoprotein cholesterol is a protective predictor of executive function in older patients with diabetes mellitus, Sun 139–146

high glucose

12(S)‐hydroxyeicosatetraenoic acid impairs vascular endothelial permeability by altering adherens junction phosphorylation levels and affecting the binding and dissociation of its components in high glucose‐induced vascular injury, Wang 639–649

histone H3 acetylation profile

Elevated histone H3 acetylation is associated with genes involved in T lymphocyte activation and glutamate decarboxylase antibody production in patients with type 1 diabetes, Wang 51–61

homeless persons

Prevalence of diabetes among homeless men in Nagoya, Japan: A survey study, Yamamoto 667–672

hyperglycemia

Novel murine model of congenital diabetes: The insulin hyposecretion mouse, Nakano 227–237
Downregulation of peroxisome proliferator‐activated receptor gamma in the placenta correlates to hyperglycemia in offspring at young adulthood after exposure to gestational diabetes mellitus, Zhao 499–512
Sustained fasting glucose oxidation and postprandial lipid oxidation associated with reduced insulin dose in type 2 diabetes with sodium–glucose cotransporter 2 inhibitor: A randomized, open‐label, prospective study, Kanazawa 1022–1031
Relationship between natural killer cell activity and glucose control in patients with type 2 diabetes and prediabetes, Kim 1223–1228
Safety of Ramadan fasting in young patients with type 1 diabetes: A systematic review and meta‐analysis, Loh 1490–1501

hyperglycemia and adverse pregnancy outcomes

Influence of different diagnostic criteria on gestational diabetes mellitus incidence and medical expenditures in China, He 1347–1357

hyperglycemic clamp

G protein‐coupled receptor 119 agonist DS‐8500a effects on pancreatic β‐cells in Japanese type 2 diabetes mellitus patients, Watada 84–93

hypertension

Contribution and interaction of the low‐density lipoprotein cholesterol to high‐density lipoprotein cholesterol ratio and triglyceride to diabetes in hypertensive patients: A cross‐sectional study, Hong 131–138
  Association of renal arteriosclerosis and hypertension with renal and cardiovascular outcomes in Japanese type 2 diabetes patients with diabetic nephropathy, Shimizu 1041–1049
Maximum morning home systolic blood pressure is an indicator of the development of diabetic nephropathy: The KAMOGAWA‐HBP study, Okamura 1543–1549

hyperuricemia

Renal impairment markers in type 2 diabetes patients with different types of hyperuricemia, Gao 118–123

hypoglycemia

Dysregulated plasma glucagon levels in Japanese young adult type 1 diabetes patients, Kawamori 62–66
Efficacy and safety of insulin glargine 300 U/mL vs insulin degludec in patients with type 2 diabetes: A randomized, open‐label, cross‐over study using continuous glucose monitoring profiles, Kawaguchi 343–351
Efficacy and safety of sitagliptin in elderly patients with type 2 diabetes mellitus: A focus on hypoglycemia, Fukuda 383–391
Clinical and enzymatic phenotypes in congenital hyperinsulinemic hypoglycemia due to glucokinase‐activating mutations: A report of two cases and a brief overview of the literature, Ping 1454–1462
Safety of Ramadan fasting in young patients with type 1 diabetes: A systematic review and meta‐analysis, Loh 1490–1501
Comparison of the efficacy and safety of insulin degludec/aspart (twice‐daily injections), insulin glargine 300 U/mL, and insulin glulisine (basal–bolus therapy), Kawaguchi 1527–1536

hypogonadism

Vitamin D_3_ supplementation improves testicular function in diabetic rats through peroxisome proliferator‐activated receptor‐γ/transforming growth factor‐beta 1/nuclear factor‐kappa B, Liu 261–271

hypoinsulinemia

Novel murine model of congenital diabetes: The insulin hyposecretion mouse, Nakano 227–237

hypoxia‐inducible factor‐1α

Macrophage‐specific hypoxia‐inducible factor‐1α deletion suppresses the development of liver tumors in high‐fat diet‐fed obese and diabetic mice, Takikawa 1411–1418

idiopathic CD4 lymphocytopenia

Evaluation of cellular and humoral autoimmunity before the development of type 1 diabetes in a patient with idiopathic CD4 lymphocytopenia, Maruyama 1108–1111

immune‐check point inhibitor

Pathogenesis of fulminant type 1 diabetes: Genes, viruses and the immune mechanism, and usefulness of patient‐derived induced pluripotent stem cells for future research, Hosokawa 1158–1164

immune‐related adverse events

Case of fulminant type 1 diabetes induced by the anti‐programmed death‐ligand 1 antibody, avelumab, Shibayama 1385–1387

immunoassay

Solid‐phase extraction treatment is required for measurement of active glucagon‐like peptide‐1 by enzyme‐linked immunosorbent assay kit affected by heterophilic antibodies, Hasegawa 302–308

induced pluripotent stem cells

Pathogenesis of fulminant type 1 diabetes: Genes, viruses and the immune mechanism, and usefulness of patient‐derived induced pluripotent stem cells for future research, Hosokawa 1158–1164

income levels

Association between income levels and irregular physician visits after a health checkup, and its consequent effect on glycemic control among employees: A retrospective propensity score‐matched cohort study, Nishi 1372–1381

inflammation

Nicotinic alpha‐7 acetylcholine receptor deficiency exacerbates hepatic inflammation and fibrosis in a mouse model of non‐alcoholic steatohepatitis, Kimura 659–666
Association between *Helicobacter pylori *infection, eradication and diabetes mellitus, Kato 1341–1346

insomnia

Association between insomnia and personality traits among Japanese patients with type 2 diabetes mellitus, Otaka 484–490

insulin degludec

Comparison of insulin glargine 300 U/mL and insulin degludec using flash glucose monitoring: A randomized cross‐over study, Yamabe 352–357

insulin gene

Identification of a variant associated with early‐onset diabetes in the intron of the insulin gene with exome sequencing, Matsuno 947–950

insulin glargine

Basal insulin therapy: Unmet medical needs in Asia and the new insulin glargine in diabetes treatment, Tien 560–570

insulin glargine 300 U/mL

Comparison of insulin glargine 300 U/mL and insulin degludec using flash glucose monitoring: A randomized cross‐over study, Yamabe 352–357

insulin‐like growth factor‐1

Insulin‐like growth factor‐1 is inversely associated with liver fibrotic markers in patients with type 2 diabetes mellitus, Miyauchi 1083–1091

insulin pump

Safety of Ramadan fasting in young patients with type 1 diabetes: A systematic review and meta‐analysis, Loh 1490–1501

insulin receptor substrate 1

p62‐mediated autophagy affects nutrition‐dependent insulin receptor substrate 1 dynamics in 3T3‐L1 preadipocytes, Igawa 32–42

insulin resistance

Activity of xanthine oxidase in plasma correlates with indices of insulin resistance and liver dysfunction in patients with type 2 diabetes mellitus and metabolic syndrome: A pilot exploratory study, Sunagawa 94–103
Effects of probiotic supplements on insulin resistance in gestational diabetes mellitus: A double‐blind randomized controlled trial, Kijmanawat 163–170
Decreased placental and muscular expression of the fibroblast growth factor 19 in gestational diabetes mellitus, Wang 171–181
Hepatocyte growth factor alleviates hepatic insulin resistance and lipid accumulation in high‐fat diet‐fed mice, Jing 251–260
Objectively measured sedentary time and diabetes mellitus in a general Japanese population: The Hisayama Study, Honda 809–816
Fasting triglycerides and glucose index is more suitable for the identification of metabolically unhealthy individuals in the Chinese adult population: A nationwide study, Yu 1050–1058
Independent links between plasma xanthine oxidoreductase activity and levels of adipokines, Furuhashi 1059–1067
Alteration of gut microbiota by a Westernized lifestyle and its correlation with insulin resistance in non‐diabetic Japanese men, Yamashita 1463–1470

insulin secretion

*In silico* and *in vitro* analyses of the pathological relevance of the R258H mutation of hepatocyte nuclear factor 4α identified in maturity‐onset diabetes of the young type 1, Sugawara 680–684

insulin sensitivity

Contribution of pancreatic α‐cell function to insulin sensitivity and glycemic variability in patients with type 1 diabetes, Takahashi 690–698

insulin signal

Protein kinase C iota facilitates insulin‐induced glucose transport by phosphorylation of soluble nSF attachment protein receptor regulator (SNARE) double C2 domain protein b, Nomiyama 591–601

insulin therapy

Development of a self‐efficacy questionnaire, ‘Insulin Therapy Self‐efficacy Scale (ITSS)’, for insulin users in Japanese: The Self‐Efficacy‐Q study, Nakaue 358–366

Insulin Therapy Self‐efficacy Scale (ITSS)

Development of a self‐efficacy questionnaire, ‘Insulin Therapy Self‐efficacy Scale (ITSS)’, for insulin users in Japanese: The Self‐Efficacy‐Q study, Nakaue 358–366

insulin‐triggered type 1 diabetes

Variants in the *BACH2* and *CLEC16A* gene might be associated with susceptibility to insulin‐triggered type 1 diabetes, Onuma 1447–1453

interaction

Independent and interactive associations of heart rate and body mass index or blood pressure with type 2 diabetes mellitus incidence: A prospective cohort study, Xu 1068–1074

International Association of Diabetes and Pregnancy Study Groups

Influence of different diagnostic criteria on gestational diabetes mellitus incidence and medical expenditures in China, He 1347–1357

intima‐media thickness

Clinical utility of carotid ultrasonography: Application for the management of patients with diabetes, Katakami 883–898

ipragliflozin

Effects of ipragliflozin on glycemic control, appetite and its related hormones: A prospective, multicenter, open‐label study (SOAR‐KOBE Study), Miura 1254–1261
Impact of body mass index on the efficacy and safety of ipragliflozin in Japanese patients with type 2 diabetes mellitus: A subgroup analysis of 3‐month interim results from the Specified Drug Use Results Survey of Ipragliflozin Treatment in Type 2 Diabetic Patients: Long‐term Use study, Tobe 1262–1271

isobornyl acrylate

Allergic contact dermatitis caused by isobornyl acrylate when using the FreeStyle^®^ Libre, Mine 1382–1384

Japan

Preferential prescribing of linagliptin in type 2 diabetes patients in an expanded post‐marketing surveillance study in Japan, Fazeli Farsani 1246–1253

Japanese

Menstrual and reproductive factors and type 2 diabetes risk: The Japan Public Health Center‐based Prospective Study, Nanri 147–153

Japanese type 2 diabetes

Verification of Kumamoto Declaration 2013 and Glycemic Targets for Elderly Patients with Diabetes in Japan for prevention of diabetic complications: A retrospective longitudinal study using outpatient clinical data, Nakanishi 290–301

JDI Updates

A new perspective on the biguanide, metformin therapy in type 2 diabetes and lactic acidosis, Hotta 906–908
The journey to understanding incretin systems: Theory, practice and more theory, Yabe 1171–1173
Diabetic kidney disease: Its current trends and future therapeutic perspectives, Koya 1174–1176
Dietary recommendations for type 2 diabetes patients: Lessons from recent clinical and basic research in Asia, Seino 1405–1407
Update on the efficacy and safety of sodium–glucose cotransporter 2 inhibitors in Asians and non‐Asians, Fujita 1408–1410

JDS/JGS guideline

Verification of Kumamoto Declaration 2013 and Glycemic Targets for Elderly Patients with Diabetes in Japan for prevention of diabetic complications: A retrospective longitudinal study using outpatient clinical data, Nakanishi 290–301

*Kbtbd11*

*Kbtbd11* gene expression in adipose tissue increases in response to feeding and affects adipocyte differentiation, Watanabe 925–932

ketoacidosis

Soluble CD163 correlates with lipid metabolic adaptations in type 1 diabetes patients during ketoacidosis, Svart 67–72

kidney disease

Protective effect of sodium–glucose cotransporter 2 inhibitors in patients with rapid renal function decline, stage G3 or G4 chronic kidney disease and type 2 diabetes, Miyoshi 1510–1517

Kumamoto Declaration

Verification of Kumamoto Declaration 2013 and Glycemic Targets for Elderly Patients with Diabetes in Japan for prevention of diabetic complications: A retrospective longitudinal study using outpatient clinical data, Nakanishi 290–301

latent autoimmune diabetes in adults

Altered T‐cell subsets and transcription factors in latent autoimmune diabetes in adults taking sitagliptin, a dipeptidyl peptidase‐4 inhibitor: A 1‐year open‐label randomized controlled trial, Wang 375–382

latent autoimmune diabetes of the adult

Glycemic variability assessed by continuous glucose monitoring and the risk of diabetic retinopathy in latent autoimmune diabetes of the adult and type 2 diabetes, Lu 753–759

leptin

Effects of ipragliflozin on glycemic control, appetite and its related hormones: A prospective, multicenter, open‐label study (SOAR‐KOBE Study), Miura 1254–1261

Letter to the Editor

“Preserved” glucagon secretion in fulminant type 1 diabetes, Murase‐Mishiba 186–187
Response to “Preserved” glucagon secretion in fulminant type 1 diabetes, Takahashi 188–189
Case of a novel PAX6 mutation with aniridia and insulin‐dependent diabetes mellitus, Motoda 552–553
Glucagon secretions are impaired in patients with fulminant type 1 diabetes, Komada 866–867
“Switched” metabolic acidosis in mitochondrial diabetes mellitus, Keidai 1116–1117
Longitudinal impact of dapagliflozin treatment on ventricular repolarization heterogeneity in patients with type 2 diabetes, Sato 1593–1594
Insulin degludec and insulin glargine 300 U/mL: Which of these two insulins causes less hypoglycemia?, Buscemi 1595–1596
Response to insulin degludec and insulin glargine 300 U/mL: Which of these two insulins causes less hypoglycemia?, Yamabe 1597–1598

lifestyle

Long working hours and skipping breakfast concomitant with late evening meals are associated with suboptimal glycemic control among young male Japanese patients with type 2 diabetes, Azami 73–83

lifestyle related diseases

Recommended configuration for personal health records by standardized data item sets for diabetes mellitus and associated chronic diseases: A report from Collaborative Initiative by six Japanese Associations, Nakashima 868–875

lifestyle Westernization

Alteration of gut microbiota by a Westernized lifestyle and its correlation with insulin resistance in non‐diabetic Japanese men, Yamashita 1463–1470

linagliptin

Preferential prescribing of linagliptin in type 2 diabetes patients in an expanded post‐marketing surveillance study in Japan, Fazeli Farsani 1246–1253

lipid abnormality

Contribution and interaction of the low‐density lipoprotein cholesterol to high‐density lipoprotein cholesterol ratio and triglyceride to diabetes in hypertensive patients: A cross‐sectional study, Hong 131–138

lipid metabolism

Hepatocyte growth factor alleviates hepatic insulin resistance and lipid accumulation in high‐fat diet‐fed mice, Jing 251–260

lipopolysaccharide

Interactive association of lipopolysaccharide and free fatty acid with the prevalence of type 2 diabetes: A community‐based cross‐sectional study, Huang 1438–1446

lipoprotein

Plasma tyrosine and its interaction with low high‐density lipoprotein cholesterol and the risk of type 2 diabetes mellitus in Chinese, Li 491–498

liver cancer

Macrophage‐specific hypoxia‐inducible factor‐1α deletion suppresses the development of liver tumors in high‐fat diet‐fed obese and diabetic mice, Takikawa 1411–1418

*LOX‐1*

Effect of oral magnesium sulfate administration on lectin‐like oxidized low‐density lipoprotein receptor‐1 gene expression to prevent atherosclerosis in diabetic rat vessels, Fazlali 650–658

M2‐like macrophages

M2‐like macrophages serve as a niche for adipocyte progenitors in adipose tissue, Nawaz 1394–1400

Macrophage

Macrophage‐specific hypoxia‐inducible factor‐1α deletion suppresses the development of liver tumors in high‐fat diet‐fed obese and diabetic mice, Takikawa 1411–1418

magnesium

Effect of oral magnesium sulfate administration on lectin‐like oxidized low‐density lipoprotein receptor‐1 gene expression to prevent atherosclerosis in diabetic rat vessels, Fazlali 650–658

manual

Diabetes Care Providers’ Manual for Disaster Diabetes Care, Satoh 1118–1142

maturity‐onset diabetes of the young

Identification and functional analysis of *GCK *gene mutations in 12 Chinese families with hyperglycemia, Wang 963–971
Pregnancy outcome of Japanese patients with glucokinase–maturity‐onset diabetes of the young, Hosokawa 1586–1589

maturity‐onset diabetes of the young type 1

*In silico* and *in vitro* analyses of the pathological relevance of the R258H mutation of hepatocyte nuclear factor 4α identified in maturity‐onset diabetes of the young type 1, Sugawara 680–684

maturity‐onset diabetes of the young type 5

Phenotypic differences and similarities of monozygotic twins with maturity‐onset diabetes of the young type 5, Ohara 1112–1115

maturity‐onset diabetes of the young type 5 during pregnancy

Maturity‐onset diabetes of the young type 5 uncovered during pregnancy with a long‐term diagnosis of type 1 diabetes, Deng 1590–1592

maximum home blood pressure

Maximum morning home systolic blood pressure is an indicator of the development of diabetic nephropathy: The KAMOGAWA‐HBP study, Okamura 1543–1549

medical costs

Retrospective analysis of medical costs and resource utilization for severe hypoglycemic events in patients with type 2 diabetes in Japan, Ikeda 857–865

medication adherence

Understanding of antidiabetic medication is associated with blood glucose in patients with type 2 diabetes: At baseline date of the KAMOGAWA‐DM cohort study, Sakai 458–465

menopause

Alendronate improves fasting plasma glucose and insulin sensitivity, and decreases insulin resistance in prediabetic osteopenic postmenopausal women: A randomized triple‐blind clinical trial, Karimi Fard 731–737

meta‐analysis

Effects of sodium–glucose cotransporter 2 inhibitors in addition to insulin therapy on cardiovascular risk factors in type 2 diabetes patients: A meta‐analysis of randomized controlled trials, Wu 446–457

metabolically unhealthy

Fasting triglycerides and glucose index is more suitable for the identification of metabolically unhealthy individuals in the Chinese adult population: A nationwide study, Yu 1050–1058

metabolic memory

Genetic variants in *DNMT1 *and the risk of cardiac autonomic neuropathy in women with type 1 diabetes, Santos‐Bezerra 985–989

metabolic syndrome

Effects of endurance training on hippocampus DJ‐1, cannabinoid receptor type 2 and blood glucose concentration in diabetic rats, Kurd 43–50
Self‐reported snoring is associated with chronic kidney disease independent of metabolic syndrome in middle‐aged and elderly Chinese, Song 124–130
Metabolic syndrome increases cardiovascular risk in a population with prediabetes: A prospective study in a cohort of Chinese adults, Chen 673–679
Clinical polyneuropathy does not increase with prediabetes or metabolic syndrome in the Japanese general population, Kurisu 1565–1575

metabolome

Exploration of predictive metabolic factors for gestational diabetes mellitus in Japanese women using metabolomic analysis, Sakurai 513–520

metformin

Which is better, high‐dose metformin monotherapy or low‐dose metformin/linagliptin combination therapy, in improving glycemic variability in type 2 diabetes patients with insufficient glycemic control despite low‐dose metformin monotherapy? A randomized, cross‐over, continuous glucose monitoring‐based pilot study, Takahashi 714–722
Effects of metformin and alogliptin on body composition in people with type 2 diabetes, Takeshita 723–730
Efficacy of metformin on postprandial plasma triglyceride concentration by administration timing in patients with type 2 diabetes mellitus: A randomized cross‐over pilot study, Sato 1284–1290

microangiopathy

Cutaneous microangiopathy in patients with type 2 diabetes: Impaired vascular endothelial growth factor expression and its correlation with neuropathy, retinopathy and nephropathy, Sugimoto 1318–1331

mirogabalin

Mirogabalin for the treatment of diabetic peripheral neuropathic pain: A randomized, double‐blind, placebo‐controlled phase III study in Asian patients, Baba 1299–1306

mobile applications

Effects of mobile phone application combined with or without self‐monitoring of blood glucose on glycemic control in patients with diabetes: A randomized controlled trial, Yu 1365–1371

moderate‐to‐vigorous‐intensity physical activity

Inverse association between physical activity and blood glucose is independent of sex, menopause status and first‐degree family history of diabetes, Hu 1502–1509

monozygotic twins

Phenotypic differences and similarities of monozygotic twins with maturity‐onset diabetes of the young type 5, Ohara 1112–1115

mutation

Identification of a variant associated with early‐onset diabetes in the intron of the insulin gene with exome sequencing, Matsuno 947–950

natural killer cell activity

Relationship between natural killer cell activity and glucose control in patients with type 2 diabetes and prediabetes, Kim 1223–1228

nausea

Clinical factors associated with the occurrence of nausea and vomiting in type 2 diabetes patients treated with glucagon‐like peptide‐1 receptor agonists, Shiomi 408–417

nephropathy

Changes in soluble tumor necrosis factor receptor type 1 levels and early renal function decline in patients with diabetes, MacIsaac 1537–1542

nerve conduction study

Validity and reliability of a point‐of‐care nerve conduction device in diabetes patients, Shibata 1291–1298

nerve conduction velocity

Aldose reductase inhibitor ranirestat significantly improves nerve conduction velocity in diabetic polyneuropathy: A randomized double‐blind placebo‐controlled study in Japan, Sekiguchi 466–474

neurodegenerative diseases

Effects of endurance training on hippocampus DJ‐1, cannabinoid receptor type 2 and blood glucose concentration in diabetic rats, Kurd 43–50

nocturnal hypoglycemia

Comparison of glucose monitoring between Freestyle Libre Pro and iPro2 in patients with diabetes mellitus, Kumagai 851–856

non‐alcoholic fatty liver disease

Serum 3‐carboxy‐4‐methyl‐5‐propyl‐2‐furanpropanoic acid is associated with lipid profiles and might protect against non‐alcoholic fatty liver disease in Chinese individuals, Dai 793–800
Effects of canagliflozin on body composition and hepatic fat content in type 2 diabetes patients with non‐alcoholic fatty liver disease, Inoue 1004–1011
Insulin‐like growth factor‐1 is inversely associated with liver fibrotic markers in patients with type 2 diabetes mellitus, Miyauchi 1083–1091

non‐alcoholic steatohepatitis

Nicotinic alpha‐7 acetylcholine receptor deficiency exacerbates hepatic inflammation and fibrosis in a mouse model of non‐alcoholic steatohepatitis, Kimura 659–666

non‐obese diabetic mouse

Novel murine model of congenital diabetes: The insulin hyposecretion mouse, Nakano 227–237

nuclear factor erythroid 2‐related factor 2

Glycine increases glyoxalase‐1 function by promoting nuclear factor erythroid 2‐related factor 2 translocation into the nucleus of kidney cells of streptozotocin‐induced diabetic rats, Wang 1189–1198

nutrient intake

Associations of nutrient intakes with obesity and diabetes mellitus in the longitudinal medical surveys of Japanese Americans, Sugihiro 1229–1236

nutritional therapy

Effect of mild exercise on glycemic and bodyweight control in Japanese type 2 diabetes patients: A retrospective analysis, Nakanishi 104–107

obesity

Glucose‐dependent insulinotropic polypeptide deficiency reduced fat accumulation and insulin resistance, but deteriorated bone loss in ovariectomized mice, Shimazu‐Kuwahara 909–914
Associations of nutrient intakes with obesity and diabetes mellitus in the longitudinal medical surveys of Japanese Americans, Sugihiro 1229–1236
Prevalence and risk factors for painful diabetic neuropathy in secondary healthcare in Qatar, Ponirakis 1558–1564

obesity disease

Brown rice‐specific γ‐oryzanol as a promising prophylactic avenue to protect against diabetes mellitus and obesity in humans, Masuzaki 18–25

omega‐3 polyunsaturated fatty acids

Omega‐3 polyunsaturated fatty acids exert anti‐oxidant effects through the nuclear factor (erythroid‐derived 2)‐related factor 2 pathway in immortalized mouse Schwann cells, Tatsumi 602–612

outcomes

Prevalence of diabetes and its effects on stroke outcomes: A meta‐analysis and literature review, Lau 780–792

outpatient clinic

Effect of mild exercise on glycemic and bodyweight control in Japanese type 2 diabetes patients: A retrospective analysis, Nakanishi 104–107

ovariectomy

Glucose‐dependent insulinotropic polypeptide deficiency reduced fat accumulation and insulin resistance, but deteriorated bone loss in ovariectomized mice, Shimazu‐Kuwahara 909–914

overweight

Weight control before and during pregnancy for patients with gestational diabetes mellitus, Yasuda 1075–1082

oxidative stress

Review of the role of cigarette smoking in diabetic foot, Xia 202–215
Omega‐3 polyunsaturated fatty acids exert anti‐oxidant effects through the nuclear factor (erythroid‐derived 2)‐related factor 2 pathway in immortalized mouse Schwann cells, Tatsumi 602–612
Canagliflozin, a sodium–glucose cotransporter 2 inhibitor, normalizes renal susceptibility to type 1 cardiorenal syndrome through reduction of renal oxidative stress in diabetic rats, Kimura 933–946

p62

p62‐mediated autophagy affects nutrition‐dependent insulin receptor substrate 1 dynamics in 3T3‐L1 preadipocytes, Igawa 32–42

pain

Mirogabalin for the treatment of diabetic peripheral neuropathic pain: A randomized, double‐blind, placebo‐controlled phase III study in Asian patients, Baba 1299–1306

painful diabetic peripheral neuropathy

Prevalence and risk factors for painful diabetic neuropathy in secondary healthcare in Qatar, Ponirakis 1558–1564

pancreatic β‐cell

Early administration of dapagliflozin preserves pancreatic β‐cell mass through a legacy effect in a mouse model of type 2 diabetes, Kanno 577–590
Contribution of pancreatic α‐cell function to insulin sensitivity and glycemic variability in patients with type 1 diabetes, Takahashi 690–698

pancreatic β‐cells

G protein‐coupled receptor 119 agonist DS‐8500a effects on pancreatic β‐cells in Japanese type 2 diabetes mellitus patients, Watada 84–93

parity

Menstrual and reproductive factors and type 2 diabetes risk: The Japan Public Health Center‐based Prospective Study, Nanri 147–153

patient medication knowledge

Understanding of antidiabetic medication is associated with blood glucose in patients with type 2 diabetes: At baseline date of the KAMOGAWA‐DM cohort study, Sakai 458–465

peptide

Leucine–glycine and carnosine dipeptides prevent diabetes induced by multiple low‐doses of streptozotocin in an experimental model of adult mice, Vahdatpour 1177–1188

peroxisome proliferator‐activated receptor gamma

Downregulation of peroxisome proliferator‐activated receptor gamma in the placenta correlates to hyperglycemia in offspring at young adulthood after exposure to gestational diabetes mellitus, Zhao 499–512

personal health record

Recommended configuration for personal health records by standardized data item sets for diabetes mellitus and associated chronic diseases: A report from Collaborative Initiative by six Japanese Associations, Nakashima 868–875

personality

Association between insomnia and personality traits among Japanese patients with type 2 diabetes mellitus, Otaka 484–490

physical activity

Effects of endurance training on hippocampus DJ‐1, cannabinoid receptor type 2 and blood glucose concentration in diabetic rats, Kurd 43–50
Assessment of energy expenditure using doubly labeled water, physical activity by accelerometer and reported dietary intake in Japanese men with type 2 diabetes: A preliminary study, Yoshimura 318–321

physical exercise

Effect of mild exercise on glycemic and bodyweight control in Japanese type 2 diabetes patients: A retrospective analysis, Nakanishi 104–107

plasma DPP‐4 activity

Effect of switching to teneligliptin from other dipeptidyl peptidase‐4 inhibitors on glucose control and renoprotection in type 2 diabetes patients with diabetic kidney disease, Kitada 706–713

plasma glucose

Inverse association between physical activity and blood glucose is independent of sex, menopause status and first‐degree family history of diabetes, Hu 1502–1509

pleiotropic effects

Effects of metformin and alogliptin on body composition in people with type 2 diabetes, Takeshita 723–730

point‐of‐care device

Validity and reliability of a point‐of‐care nerve conduction device in diabetes patients, Shibata 1291–1298

*Polygonatum sibiricum* polysaccharide

*Polygonatum sibiricum* polysaccharide potentially attenuates diabetic retinal injury in a diabetic rat model, Wang 915–924

postmarketing product surveillance

Impact of body mass index on the efficacy and safety of ipragliflozin in Japanese patients with type 2 diabetes mellitus: A subgroup analysis of 3‐month interim results from the Specified Drug Use Results Survey of Ipragliflozin Treatment in Type 2 Diabetic Patients: Long‐term Use study, Tobe 1262–1271

post‐marketing study

Safety and efficacy of tofogliflozin in Japanese patients with type 2 diabetes mellitus in real‐world clinical practice: Results of 3‐month interim analysis of a long‐term post‐marketing surveillance study (J‐STEP/LT), Utsunomiya 1272–1283

postprandial hyperglycemia

Comparison of glucose monitoring between Freestyle Libre Pro and iPro2 in patients with diabetes mellitus, Kumagai 851–856

postprandial hypertriglyceridemia

Efficacy of metformin on postprandial plasma triglyceride concentration by administration timing in patients with type 2 diabetes mellitus: A randomized cross‐over pilot study, Sato 1284–1290

prediabetes

Metabolic syndrome increases cardiovascular risk in a population with prediabetes: A prospective study in a cohort of Chinese adults, Chen 673–679
Cardiometabolic risk factors in Thai individuals with prediabetes treated in a high‐risk, prevention clinic: Unexpected relationship between high‐density lipoprotein cholesterol and glycemia in men, Srivanichakorn 771–779
Clinical polyneuropathy does not increase with prediabetes or metabolic syndrome in the Japanese general population, Kurisu 1565–1575

prediabetic

Alendronate improves fasting plasma glucose and insulin sensitivity, and decreases insulin resistance in prediabetic osteopenic postmenopausal women: A randomized triple‐blind clinical trial, Karimi Fard 731–737

pregnancy

Pregnancy outcome of Japanese patients with glucokinase–maturity‐onset diabetes of the young, Hosokawa 1586–1589

premixed insulin twice‐daily injection therapy

Comparison of the efficacy and safety of insulin degludec/aspart (twice‐daily injections), insulin glargine 300 U/mL, and insulin glulisine (basal–bolus therapy), Kawaguchi 1527–1536

prevalence

Prevalence of gestational diabetes mellitus in mainland China: A systematic review and meta‐analysis, Gao 154–162
Associations between stressful life events and diabetes: Findings from the China Kadoorie Biobank study of 500,000 adults, Wang 1215–1222
High, but stable, trend in the prevalence of gestational diabetes mellitus: A population‐based study in Xiamen, China, Yan 1358–1364

prevalence of symmetric sensorimotor polyneuropathy

Clinical polyneuropathy does not increase with prediabetes or metabolic syndrome in the Japanese general population, Kurisu 1565–1575

probiotics

Effects of probiotic supplements on insulin resistance in gestational diabetes mellitus: A double‐blind randomized controlled trial, Kijmanawat 163–170

(pro)renin receptor

(Pro)renin receptor: Involvement in diabetic retinopathy and development of molecular targeted therapy, Kanda 6–17

protein kinase

Protein kinase C and protein kinase A are involved in the protection of recombinant human glucagon‐like peptide‐1 on glomeruli and tubules in diabetic rats, Yin 613–625

psychological factor

Novel psychosocial factor involved in diabetes self‐care in the Japanese cultural context, Mano 1102–1107

radioimmunoassay

Actual condition survey regarding mismatch of measurements between radioimmunoassay and enzyme‐linked immunosorbent assay tests for anti‐glutamic acid decarboxylase antibody in real‐world clinical practice, Oikawa 685–689

ranirestat

Aldose reductase inhibitor ranirestat significantly improves nerve conduction velocity in diabetic polyneuropathy: A randomized double‐blind placebo‐controlled study in Japan, Sekiguchi 466–474

rapid‐acting insulin analog

Assessment of optimal insulin administration timing for standard carbohydrates‐rich meals using continuous glucose monitoring in children with type 1 diabetes: A cross‐over randomized study, Tucholski 1237–1245

real‐world practice

Incidence and risk of vaginal candidiasis associated with sodium–glucose cotransporter 2 inhibitors in real‐world practice for women with type 2 diabetes, Yokoyama 439–445

receptor‐associated prorenin system

(Pro)renin receptor: Involvement in diabetic retinopathy and development of molecular targeted therapy, Kanda 6–17

recombinant human glucagon‐like peptide‐1

Protein kinase C and protein kinase A are involved in the protection of recombinant human glucagon‐like peptide‐1 on glomeruli and tubules in diabetic rats, Yin 613–625

regenerative medicine

Conditioned media from dental pulp stem cells improved diabetic polyneuropathy through anti‐inflammatory, neuroprotective and angiogenic actions: Cell‐free regenerative medicine for diabetic polyneuropathy, Makino 1199–1208

renal arteriosclerosis

Association of renal arteriosclerosis and hypertension with renal and cardiovascular outcomes in Japanese type 2 diabetes patients with diabetic nephropathy, Shimizu 1041–1049

renal impairment

Renal impairment markers in type 2 diabetes patients with different types of hyperuricemia, Gao 118–123

repaglinide

Reduction in glucose fluctuations in elderly patients with type 2 diabetes using repaglinide: A randomized controlled trial of repaglinide vs sulfonylurea, Omori 367–374

resistance training

Effects of low‐intensity resistance training on muscular function and glycemic control in older adults with type 2 diabetes, Takenami 331–338

retrospective analysis

Retrospective analysis of medical costs and resource utilization for severe hypoglycemic events in patients with type 2 diabetes in Japan, Ikeda 857–865

sarcopenia

Importance of physical evaluation using skeletal muscle mass index and body fat percentage to prevent sarcopenia in elderly Japanese diabetes patients, Fukuoka 322–330
Association of accumulated advanced glycation end‐products with a high prevalence of sarcopenia and dynapenia in patients with type 2 diabetes, Mori 1332–1340
Hyperglycemia in non‐obese patients with type 2 diabetes is associated with low muscle mass: The Multicenter Study for Clarifying Evidence for Sarcopenia in Patients with Diabetes Mellitus, Sugimoto 1471–1479

sedentary behaviors

Objectively measured sedentary time and diabetes mellitus in a general Japanese population: The Hisayama Study, Honda 809–816

self‐efficacy

Development of a self‐efficacy questionnaire, ‘Insulin Therapy Self‐efficacy Scale (ITSS)’, for insulin users in Japanese: The Self‐Efficacy‐Q study, Nakaue 358–366

self‐management

Comparison of glucose monitoring between Freestyle Libre Pro and iPro2 in patients with diabetes mellitus, Kumagai 851–856
Novel psychosocial factor involved in diabetes self‐care in the Japanese cultural context, Mano 1102–1107

self‐monitoring of blood glucose

Effects of mobile phone application combined with or without self‐monitoring of blood glucose on glycemic control in patients with diabetes: A randomized controlled trial, Yu 1365–1371

serum albumin

Efficacy and safety of insulin glargine 300 U/mL vs insulin degludec in patients with type 2 diabetes: A randomized, open‐label, cross‐over study using continuous glucose monitoring profiles, Kawaguchi 343–351

serum creatinine

Low serum creatinine and risk of diabetes: The Japan Epidemiology Collaboration on Occupational Health Study, Hu 1209–1214

severe hypoglycemia

Retrospective analysis of medical costs and resource utilization for severe hypoglycemic events in patients with type 2 diabetes in Japan, Ikeda 857–865

sex difference

Inverse association between physical activity and blood glucose is independent of sex, menopause status and first‐degree family history of diabetes, Hu 1502–1509

single‐nucleotide polymorphism

Clinical and genetic characteristics of abnormal glucose tolerance in Japanese women in the first year after gestational diabetes mellitus, Kasuga 817–826

sitagliptin

Efficacy and safety of sitagliptin in elderly patients with type 2 diabetes mellitus: A focus on hypoglycemia, Fukuda 383–391

skeletal muscle

Sodium–glucose cotransporter 2 inhibitor‐induced changes in body composition and simultaneous changes in metabolic profile: 52‐week prospective LIGHT (Luseogliflozin: the Components of Weight Loss in Japanese Patients with Type 2 Diabetes Mellitus) Study, Sasaki 108–117
Low serum creatinine and risk of diabetes: The Japan Epidemiology Collaboration on Occupational Health Study, Hu 1209–1214

skeletal muscle mass

Hyperglycemia in non‐obese patients with type 2 diabetes is associated with low muscle mass: The Multicenter Study for Clarifying Evidence for Sarcopenia in Patients with Diabetes Mellitus, Sugimoto 1471–1479

skin

Cutaneous microangiopathy in patients with type 2 diabetes: Impaired vascular endothelial growth factor expression and its correlation with neuropathy, retinopathy and nephropathy, Sugimoto 1318–1331

snoring

Self‐reported snoring is associated with chronic kidney disease independent of metabolic syndrome in middle‐aged and elderly Chinese, Song 124–130

social support

Prevalence of diabetes among homeless men in Nagoya, Japan: A survey study, Yamamoto 667–672

sodium–glucose

Protective effect of sodium–glucose cotransporter 2 inhibitors in patients with rapid renal function decline, stage G3 or G4 chronic kidney disease and type 2 diabetes, Miyoshi 1510–1517

sodium–glucose cotransporter 2 inhibitor

Safety and tolerability of empagliflozin in East Asian patients with type 2 diabetes: Pooled analysis of phase I–III clinical trials, Yabe 418–428
Should sulfonylurea be discontinued or maintained at the lowest dose when starting ipragliflozin? A multicenter observational study in Japanese patients with type 2 diabetes, Takahashi 429–438
Effects of sodium–glucose cotransporter 2 inhibitors in addition to insulin therapy on cardiovascular risk factors in type 2 diabetes patients: A meta‐analysis of randomized controlled trials, Wu 446–457
Early administration of dapagliflozin preserves pancreatic β‐cell mass through a legacy effect in a mouse model of type 2 diabetes, Kanno 577–590
Canagliflozin, a sodium–glucose cotransporter 2 inhibitor, normalizes renal susceptibility to type 1 cardiorenal syndrome through reduction of renal oxidative stress in diabetic rats, Kimura 933–946
Effects of canagliflozin on body composition and hepatic fat content in type 2 diabetes patients with non‐alcoholic fatty liver disease, Inoue 1004–1011
Ipragliflozin, a sodium–glucose cotransporter 2 inhibitor, reduces bodyweight and fat mass, but not muscle mass, in Japanese type 2 diabetes patients treated with insulin: A randomized clinical trial, Inoue 1012–1021
Sustained fasting glucose oxidation and postprandial lipid oxidation associated with reduced insulin dose in type 2 diabetes with sodium–glucose cotransporter 2 inhibitor: A randomized, open‐label, prospective study, Kanazawa 1022–1031
Long‐term safety and efficacy of the sodium–glucose cotransporter 2 inhibitor, tofogliflozin, added on glucagon‐like peptide‐1 receptor agonist in Japanese patients with type 2 diabetes mellitus: A 52‐week open‐label, multicenter, post‐marketing clinical study, Terauchi 1518–1526

sodium–glucose cotransporter 2 inhibitors

Incidence and risk of vaginal candidiasis associated with sodium–glucose cotransporter 2 inhibitors in real‐world practice for women with type 2 diabetes, Yokoyama 439–445

solid phase extraction

Solid‐phase extraction treatment is required for measurement of active glucagon‐like peptide‐1 by enzyme‐linked immunosorbent assay kit affected by heterophilic antibodies, Hasegawa 302–308

soluble CD163

Soluble CD163 correlates with lipid metabolic adaptations in type 1 diabetes patients during ketoacidosis, Svart 67–72

spatial analysis

Type 2 diabetes mellitus and neighborhood deprivation index: A spatial analysis in Zhejiang, China, Zhang 272–282

steroid‐induced hyperglycemia

Feasibility and safety of using an automated decision support system for insulin therapy in the treatment of steroid‐induced hyperglycemia in patients with acute graft‐versus‐host disease: A randomized trial, Aberer 339–342

stressful life events

Associations between stressful life events and diabetes: Findings from the China Kadoorie Biobank study of 500,000 adults, Wang 1215–1222

stroke

Prevalence of diabetes and its effects on stroke outcomes: A meta‐analysis and literature review, Lau 780–792

sulfonylurea

Efficacy and safety of sitagliptin in elderly patients with type 2 diabetes mellitus: A focus on hypoglycemia, Fukuda 383–391
Should sulfonylurea be discontinued or maintained at the lowest dose when starting ipragliflozin? A multicenter observational study in Japanese patients with type 2 diabetes, Takahashi 429–438

T cells

Evaluation of cellular and humoral autoimmunity before the development of type 1 diabetes in a patient with idiopathic CD4 lymphocytopenia, Maruyama 1108–1111

teneligliptin

Effect of switching to teneligliptin from other dipeptidyl peptidase‐4 inhibitors on glucose control and renoprotection in type 2 diabetes patients with diabetic kidney disease, Kitada 706–713

thinness

Being underweight in adolescence is independently associated with adult‐onset diabetes among women: The Japan Nurses’ Health Study, Katanoda 827–836

T‐lymphocyte subsets

Altered T‐cell subsets and transcription factors in latent autoimmune diabetes in adults taking sitagliptin, a dipeptidyl peptidase‐4 inhibitor: A 1‐year open‐label randomized controlled trial, Wang 375–382

TNF receptors

Changes in soluble tumor necrosis factor receptor type 1 levels and early renal function decline in patients with diabetes, MacIsaac 1537–1542

tofogliflozin

Safety and efficacy of tofogliflozin in Japanese patients with type 2 diabetes mellitus in real‐world clinical practice: Results of 3‐month interim analysis of a long‐term post‐marketing surveillance study (J‐STEP/LT), Utsunomiya 1272–1283

total energy expenditure

Assessment of energy expenditure using doubly labeled water, physical activity by accelerometer and reported dietary intake in Japanese men with type 2 diabetes: A preliminary study, Yoshimura 318–321

treatment

Glucagon‐like peptide‐1 receptor agonists for type 2 diabetes: A rational drug development, Ahrén 196–201

treatment drug

Ipragliflozin, a sodium–glucose cotransporter 2 inhibitor, reduces bodyweight and fat mass, but not muscle mass, in Japanese type 2 diabetes patients treated with insulin: A randomized clinical trial, Inoue 1012–1021

treatment satisfaction

Improvement in treatment satisfaction after switching from liraglutide to dulaglutide in patients with type 2 diabetes: A randomized controlled trial, Takase 699–705

triglycerides

Serum 3‐carboxy‐4‐methyl‐5‐propyl‐2‐furanpropanoic acid is associated with lipid profiles and might protect against non‐alcoholic fatty liver disease in Chinese individuals, Dai 793–800

triglycerides and glucose index

Fasting triglycerides and glucose index is more suitable for the identification of metabolically unhealthy individuals in the Chinese adult population: A nationwide study, Yu 1050–1058

tubular injury

Pathological significance of urinary complement activation in diabetic nephropathy: A full view from the development of the disease, Zheng 738–744

type 1 diabetes

Elevated histone H3 acetylation is associated with genes involved in T lymphocyte activation and glutamate decarboxylase antibody production in patients with type 1 diabetes, Wang 51–61
Dysregulated plasma glucagon levels in Japanese young adult type 1 diabetes patients, Kawamori 62–66
Contribution of pancreatic α‐cell function to insulin sensitivity and glycemic variability in patients with type 1 diabetes, Takahashi 690–698
Evaluation of cellular and humoral autoimmunity before the development of type 1 diabetes in a patient with idiopathic CD4 lymphocytopenia, Maruyama 1108–1111
Assessment of optimal insulin administration timing for standard carbohydrates‐rich meals using continuous glucose monitoring in children with type 1 diabetes: A cross‐over randomized study, Tucholski 1237–1245
Circulating anti‐glutamic acid decarboxylase‐65 antibody titers are positively associated with the capacity of insulin secretion in acute‐onset type 1 diabetes with short duration in a Japanese population, Yamamura 1480–1489

type 2 diabetes

Long working hours and skipping breakfast concomitant with late evening meals are associated with suboptimal glycemic control among young male Japanese patients with type 2 diabetes, Azami 73–83
Menstrual and reproductive factors and type 2 diabetes risk: The Japan Public Health Center‐based Prospective Study, Nanri 147–153
Glucagon‐like peptide‐1 receptor agonists for type 2 diabetes: A rational drug development, Ahrén 196–201
Dietary survey in Japanese patients with type 2 diabetes and the influence of dietary carbohydrate on glycated hemoglobin: The Sleep and Food Registry in Kanagawa study, Yamakawa 309–317
Plasma tyrosine and its interaction with low high‐density lipoprotein cholesterol and the risk of type 2 diabetes mellitus in Chinese, Li 491–498
Direct medical costs for patients with type 2 diabetes in 16 tertiary hospitals in urban China: A multicenter prospective cohort study, Li 539–551
Improvement in treatment satisfaction after switching from liraglutide to dulaglutide in patients with type 2 diabetes: A randomized controlled trial, Takase 699–705
Key genes and co‐expression network analysis in the livers of type 2 diabetes patients, Li 951–962
Sustained fasting glucose oxidation and postprandial lipid oxidation associated with reduced insulin dose in type 2 diabetes with sodium–glucose cotransporter 2 inhibitor: A randomized, open‐label, prospective study, Kanazawa 1022–1031
Preferential prescribing of linagliptin in type 2 diabetes patients in an expanded post‐marketing surveillance study in Japan, Fazeli Farsani 1246–1253
Interactive association of lipopolysaccharide and free fatty acid with the prevalence of type 2 diabetes: A community‐based cross‐sectional study, Huang 1438–1446
Hyperglycemia in non‐obese patients with type 2 diabetes is associated with low muscle mass: The Multicenter Study for Clarifying Evidence for Sarcopenia in Patients with Diabetes Mellitus, Sugimoto 1471–1479
Prevalence and risk factors for painful diabetic neuropathy in secondary healthcare in Qatar, Ponirakis 1558–1564

type 2 diabetes mellitus

Renal impairment markers in type 2 diabetes patients with different types of hyperuricemia, Gao 118–123
Catastrophic health expenditure among type 2 diabetes mellitus patients: A province‐wide study in Shandong, China, Jing 283–289
Assessment of energy expenditure using doubly labeled water, physical activity by accelerometer and reported dietary intake in Japanese men with type 2 diabetes: A preliminary study, Yoshimura 318–321
Effects of liraglutide, metformin and gliclazide on body composition in patients with both type 2 diabetes and non‐alcoholic fatty liver disease: A randomized trial, Feng 399–407
Association between insomnia and personality traits among Japanese patients with type 2 diabetes mellitus, Otaka 484–490
Early administration of dapagliflozin preserves pancreatic β‐cell mass through a legacy effect in a mouse model of type 2 diabetes, Kanno 577–590
Empagliflozin and kidney outcomes in Asian patients with type 2 diabetes and established cardiovascular disease: Results from the EMPA‐REG OUTCOME^®^ trial, Kadowaki 760–770
Association of gamma‐glutamyl transferase and alanine aminotransferase with type 2 diabetes mellitus incidence in middle‐aged Japanese men: 12‐year follow up, Kaneko 837–845
Independent and interactive associations of heart rate and body mass index or blood pressure with type 2 diabetes mellitus incidence: A prospective cohort study, Xu 1068–1074
Insulin‐like growth factor‐1 is inversely associated with liver fibrotic markers in patients with type 2 diabetes mellitus, Miyauchi 1083–1091
Relationship between natural killer cell activity and glucose control in patients with type 2 diabetes and prediabetes, Kim 1223–1228
Safety and efficacy of tofogliflozin in Japanese patients with type 2 diabetes mellitus in real‐world clinical practice: Results of 3‐month interim analysis of a long‐term post‐marketing surveillance study (J‐STEP/LT), Utsunomiya 1272–1283
Lower body mass index is not of more benefit for diabetic complications, Zhang 1307–1317
Long‐term safety and efficacy of the sodium–glucose cotransporter 2 inhibitor, tofogliflozin, added on glucagon‐like peptide‐1 receptor agonist in Japanese patients with type 2 diabetes mellitus: A 52‐week open‐label, multicenter, post‐marketing clinical study, Terauchi 1518–1526

uric acid

Activity of xanthine oxidase in plasma correlates with indices of insulin resistance and liver dysfunction in patients with type 2 diabetes mellitus and metabolic syndrome: A pilot exploratory study, Sunagawa 94–103
Independent links between plasma xanthine oxidoreductase activity and levels of adipokines, Furuhashi 1059–1067

urinary complement activation products

Pathological significance of urinary complement activation in diabetic nephropathy: A full view from the development of the disease, Zheng 738–744

vaginal candidiasis

Incidence and risk of vaginal candidiasis associated with sodium–glucose cotransporter 2 inhibitors in real‐world practice for women with type 2 diabetes, Yokoyama 439–445

vascular complications

Lower body mass index is not of more benefit for diabetic complications, Zhang 1307–1317

vascular endothelial growth factor

Clinical preferences and trends of anti‐vascular endothelial growth factor treatments for diabetic macular edema in Japan, Sugimoto 475–483
Cutaneous microangiopathy in patients with type 2 diabetes: Impaired vascular endothelial growth factor expression and its correlation with neuropathy, retinopathy and nephropathy, Sugimoto 1318–1331

vildagliptin

Drug fever and acute inflammation from hypercytokinemia triggered by dipeptidyl peptidase‐4 inhibitor vildagliptin, Anno 182–185

visceral fat

Sodium–glucose cotransporter 2 inhibitor‐induced changes in body composition and simultaneous changes in metabolic profile: 52‐week prospective LIGHT (Luseogliflozin: the Components of Weight Loss in Japanese Patients with Type 2 Diabetes Mellitus) Study, Sasaki 108–117

vomiting

Clinical factors associated with the occurrence of nausea and vomiting in type 2 diabetes patients treated with glucagon‐like peptide‐1 receptor agonists, Shiomi 408–417

weighted gene correlation network analysis

Key genes and co‐expression network analysis in the livers of type 2 diabetes patients, Li 951–962

white adipocytes

Fat‐specific protein 27α inhibits autophagy‐dependent lipid droplet breakdown in white adipocytes, Nakajima 1419–1429

xanthine oxidase

Activity of xanthine oxidase in plasma correlates with indices of insulin resistance and liver dysfunction in patients with type 2 diabetes mellitus and metabolic syndrome: A pilot exploratory study, Sunagawa 94–103

